# Health Reforms Should Focus on Improving Services and Systems, Not Just Containing Costs

**DOI:** 10.3389/ijph.2021.1604332

**Published:** 2021-12-29

**Authors:** Aron Baumann

**Affiliations:** ^1^ Swiss Tropical and Public Health Institute (Swiss TPH), Basel, Switzerland; ^2^ University of Basel, Basel, Switzerland

**Keywords:** healthcare expenditure, quality of care, health reform, cost containment, inefficient healthcare

**The IJPH series “Young Researcher Editorial” is a training project of the Swiss School of Public Health.**

Health systems must continuously evolve and strive for efficiency both in care and its organization to meet the challenges of society, keep pace with medical progress and cope with rising costs [1]. In the absence of adequate protection systems, rising costs may threaten health equity, social justice, and health outcomes [2]. Policy measures for containing costs (by stabilizing health expenditures or slowing growth) may make health systems more financially sustainable and reduce the cost of care, but they may be ineffective by design and result in negative consequences on health [3]. Instead of simply focusing on reducing costs, health reforms should aim to increase efficiency.

For example, if policies to rigidly contain costs are introduced in response to an economic crisis, patients may have a harder time accessing healthcare, suicide rates may rise, and infectious disease outbreaks may increase [2]. When governments take measures to reduce budget deficits, they can worsen social determinants and indicators of child health [4]. Policy measures designed to cut costs in the short-term, but that do not make sustainable changes in the structure and organization of the health system may make systems less responsive and neglect investments in, e.g., prevention which can save money over the long-term [5, 6]. Cutting necessary services, restricting health insurance coverage, and increasing cost-sharing can reduce the likelihood governments will meet health targets and worsen health outcomes.

A recent review of the United States’ health system found waste constituted about a quarter of total health care expenditures [7]. Reforms designed to contain costs should ideally curb ineffective or wasteful health services and bureaucratic functions and have positive outcomes like improving delivery and coordinating care, reducing overtreatment, or simplifying unnecessarily complex administrative systems [8]. Costs could also be saved by reducing unnecessary care by, for example, shifting costly inpatient services to the outpatient sector [9].

But reforming the health system to increase its efficiency requires coping with fragmented governance structures and stakeholder interests [7, 10]. These challenges must be addressed when introducing alternative provider payment methods, centralizing procurement, reducing drug prices, or reconfiguring services toward integrative care. Such attempts may be impeded by policy actors with strong veto powers. Federalized or decentralized policy structures may make it hard to make and implement decisions when responsibility is distributed across policy levels and actors [5, 6]. In these types of systems, it may be helpful to use evidence to draw attention to problem areas that are generally accepted as relevant to action, and to target reforms accordingly.

Focusing reforms on problem areas in the health system requires consideration of two main factors. First, policy makers must be able to draw on comprehensive quality and outcome data that demonstrate inefficiencies in care, and integrate this evidence into arguments for reform. These data should be translated into monetary terms (e.g., through economic evaluations) so policy makers can argue for value-based health services and make persuasive arguments for policy change. This data is best collected from and processed within interoperable electronic systems that enable exchange between stakeholders. Establishing and applying quality indicators by, for example, including patient-reported outcome and experience measures, can help identify fields of action. Developing strategies and providing resources to collect and apply quality of care data can contain costs over the long term. Along with collecting data crucial to monitoring public health, governments must support and co-produce research on health services and the health system so policy makers can compare health service utilization, performance, and outcomes within and between countries to identify policy solutions.

Second, policy actors like health authorities must be able to request and collect data, consult relevant research literature, and develop and implement evidence-informed interventions to address current problems with health service provision and organization. Health services can be managed proactively and their inefficiencies addressed more consistently if health authorities are adequately staffed and can translate available evidence into policy decisions. Policy makers who engage with evidence can more quickly take advantage of emerging policy windows, choose and frame acceptable health system improvement measures, and collaborate with other stakeholders to inform and build majorities for policy alternatives even when faced with political resistance.

Reforms designed to increase the quality of a health system and reduce its inefficiencies will have better long-term outcomes than reforms that focus only on cost savings. But if these reforms are to be successful, we must make it easier to collect and analyze quality and outcome data and support health authorities to consult more health services research. One step in this direction is that governments strengthen health authorities' resources and abilities to proactively engage with health services and system evidence to inform policy development for effective and efficient solutions.
